# Association between clinical use of lansoprazole and the risk of type 2 diabetes mellitus: a pharmacoepidemiological cohort study

**DOI:** 10.1186/s13098-023-01051-8

**Published:** 2023-05-10

**Authors:** Ming-Hsun Lin, Wen‐Tung Wu, Yong-Chen Chen, Tsung-Kun Lin, Yu‐Ching Chou, Chien‐An Sun

**Affiliations:** 1 Division of Endocrinology and Metabolism, Department of Internal Medicine, Tri-Service General Hospital, National Defense Medical Center, Taipei City, 114 Taiwan; 2 Department of Pharmacy, Tri-Service General Hospital, National Defense Medical Center, Taipei City, 114 Taiwan; 3 Department of Public Health, College of Medicine, Fu-Jen Catholic University, No.510, Zhongzheng Road, Xinzhuang District, New Taipei City, 242 Taiwan; 4 Department of Medicine, College of Medicine, Fu-Jen Catholic University, New Taipei City, 242 Taiwan; 5 School of Pharmacy, National Defense Medical Center, Taipei City, 114 Taiwan; 6 School of Public Health, National Defense Medical Center, Taipei City, 114 Taiwan

**Keywords:** Cohort studies, Lansoprazole, Proton pump inhibitors, Type, 2 diabetes mellitus

## Abstract

**Background:**

Proton pump inhibitors (PPIs) are common and widely used for gastrointestinal-related disorders. Lansoprazole is one of PPIs with potential benefits of anti-inflammation, reduced oxidative stress, and anti-diabetes. The aims of this study are to determine whether lansoprazole imparts differential risk of type 2 diabetes as compared with other PPIs.

**Methods:**

A population-based retrospective cohort study was conducted using the National Health Insurance Research Database in Taiwan. Patients who received lansoprazole more than 90 days and without records of use of other PPIs between January 1, 2000 and December 31, 2005 (the exposure period) were considered as the exposed cohort (n = 1668). In comparison, patients who received other PPIs more than 90 days and without use of lansoprazole in the exposure period were treated as the comparison cohort (n = 3336).The primary outcome was the new-onset of type 2 diabetes mellitus (T2DM). The association between use of lansoprazole and the risk of T2DM was determined by hazard ratios (HRs) and 95% confidence intervals (CIs) derived from multivariable Cox proportional hazards models.

**Results:**

The lansoprazole cohort showed a significantly reduced risk of T2DM with an adjusted HR of 0.65 (95% CI 0.56–0.76). Interestingly, the inverse association between use of lansoprazole and risk of T2DM was observed in both genders and in various age groups.

**Conclusion:**

The present study findings suggest that lansoprazole was associated with a reduced risk of T2DM compared with other PPIs. Further studies are needed to determine the clinical implications of the present study.

## Introduction

Type 2 diabetes mellitus (T2DM) is recognized as a serious public health concern with a considerable impact on human life and health expenditures. Globally, there were 437.9 million prevalent cases of T2DM in 2019, with an age-adjusted prevalence rate of 5282.9 per 100,000 populations, which has increased 10.8% since 1990 [1]. Remarkably, certain regions, such as Southeast Asian countries including Indonesia, Malaysia, Thailand, and Vietnam, have moved up the ranks in the last two decades. In particular, owing to their large population sizes, China (88.5 million individuals with type 2 diabetes) and India (65.9 million) retain the top spots as the countries with the greatest total number of individuals with this condition [2].It has been noted that a high prevalence rate of upper gastrointestinal disorders was observed in patients with T2DM, especially among those with poor glycemic controls [3]. Proton pump inhibitors (PPIs) are widely used as acid inhibitory agents for the treatment of gastroesophageal-related disorders [4]. By blocking H + /K + ATPase, PPIs are potent gastric acid inhibitors [4]. However, PPIs do elevate gastrin levels through negative feedback [5]. In vitro studies have indicated that gastrin induces β cell neogenesis and increases β cell mass [6, 7]. In addition, several observational studies [8, 9] and randomized controlled trials [10, 11] have demonstrated that PPI decreased glycosylated hemoglobin (HbA1c) levels. However, evidence about the risk of T2DM associated with treatment of PPI is inconclusive [12–14]. Further, insufficient data exist regarding the effects of PPI on diabetic risk among Asian populations.

Lansoprazole(LPZ) is an effective and popular PPIs with few side effects [15]. It is different from other PPIs with regards to pharmacological properties of potential inflammatory inhibition, reduced oxidative stress, and anticancer potentials [16–18]. As such, the aims of this investigation are to determine whether use of lansoprazole imparts differential risk of T2DM as compared with other PPIs. Hence, we conducted a population-based retrospective cohort study to explore the relationship of lansoprazole with the risk of new-onset T2DM using data from the Taiwan National Health Insurance Research Database (NHIRD).

## Methods

### Data source

The current study was a population-based retrospective cohort study using medical 85 claims dataset from the National Health Insurance (NHI) Program in Taiwan, the NHIRD. The NHI is a publicly funded single-payer health insurance program for all Taiwanese residents. The NHIRD contains comprehensive medical care information, including demographic data of insured individuals, data of clinical visits, diagnostic codes in the format of International Classification of Diseases, Ninth revision, Clinical Modification (ICD-9- CM), and prescription details, as described previously [19]. The NHIRD can deliver as a basis for the procurement of high-quality epidemiological studies [20] with a good validity on information regarding diagnoses and drug prescription [21, 22]. The data of this study was obtained from the Longitudinal Health Insurance Database 2000 (LHID 2000), a subset of NHIRD. The LHID 2000 data set contains historical ambulatory and inpatient care data for 1 million randomly sampled beneficiaries enrolled in the NHI system in 2000. There were no significant differences in the distributions of age, sex, and healthcare costs between the individuals in LHID and NHIRD [19]. Since the data set was released for research purposes and included only scrambled information on patient identification, the study was exempt from informed consent from the subjects. Meanwhile, the present study has been approved by the Institutional Review Board of Fu-Jen Catholic University (FJU-IRB No:C104014).

### Study population

**Fig. 1:**
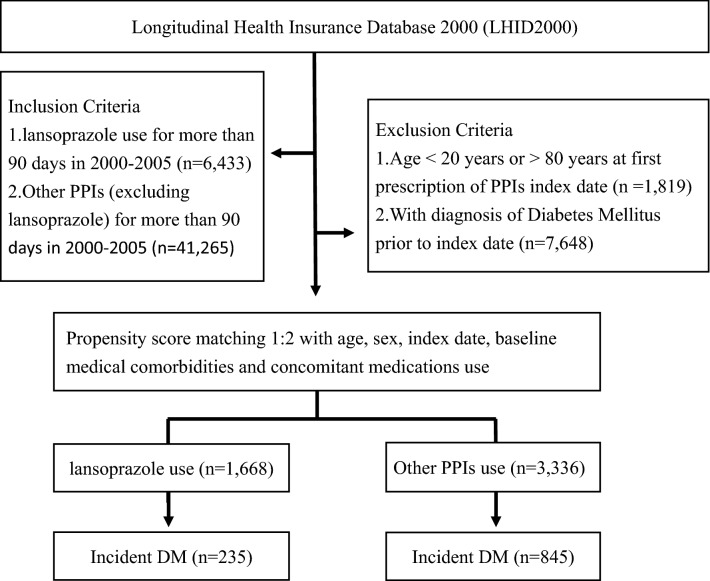
Flow chart of the study design

The source population was patients who were randomly selected into LHID 2000 and were aged between 20 and 80 years from January 1, 2000 to December 31, 2005. To be eligible, patients needed to be continuously covered by NHI between 2000 and 2005. In the present study, we used incident user design to define lansoprazole and other PPIs (including omeprazole, esomeprazole, pantoprazole, and rabeprazole) exposures. That is, patients who did undergo lansoprazole or other PPIs treatments prior to 2000 were excluded. Patients who received lansoprazole≧90 days and without any co-prescription of other PPIs between January 1, 2000 and December 31, 2005 (the exposure period) were considered as the exposed cohort. In comparison, patients who received other PPIs prescriptions≧90 days and without use of lansoprazole in the study period were considered as the comparison cohort. In this study, we used patients who received lansoprazole or other PPIs≧90 days (equal to three times of prescriptions in outpatient visits) in the study period as the stable lansoprazole or PPIs users. Patients who received lansoprazole or other PPIs with less than 90 days in the study period were excluded. In the current study, the temporal period associated with drug exposures was referred to the exposure period, which was equal for each patient. The date of initial prescriptions of lansoprazole for each patient was assigned as their index date. Initiation was defined as being free from any lansoprazole therapy for 12 months prior to the first prescription (index date). Patients in both exposed and comparison cohorts had no diagnosis of T2DM or prescriptions of anti-diabetic agents prior to the index date. To control for potential confounders between the two cohorts, we applied propensity score matching at a ratio of 1:2 for exposed and comparison cohorts. The propensity score was calculated for each patient by using a logistic regression model with covariates of age, sex, index date, baseline comorbidities, including heart failure (ICD-9-CM code 428.0), malignant neoplasms (ICD-9-CM codes:140-208), cardiovascular disease (ICD- 9-CM codes: 410-414, 425, 428, 674, and 678), hypertension (ICD-9-CM codes:401-405), hyperlipidemia (ICD-9-CM code:272.4), chronic liver disease (ICD-9-CM codes 570-572), and chronic kidney disease (ICD-9-CM code:585) and use of co-medications, including beta blocking agents (Anatomic Therapeutic Chemical (ATC) code: C07), statins (ATC codes:C10AA01, C10AA02, C10AA03, C10AA04, C10AA05, and C10AA07), corticosteroids (ATC code: R01AD), and thiazide (ATC codes:C03AA03). Cohort members were excluded from the study if they were aged < 20 years or > 80 years (n = 1819), had been diagnosed with T2DM and/or use of anti-diabetic agents prior to the index date (n = 7648). We finally included 1,668 patients as the exposed cohort and 3,336 patients as the comparison cohort (Fig. 1). All of the study participants were followed from the index date to the onset of T2DM, death (as indicated by disenrollment from the NHI) or the end of the study date (December 31, 2013), whichever occurred first.

### Outcome determination

The primary outcome was the occurrence of new-onset T2DM. To ensure the diagnostic validity of T2DM, we determined patients having one hospital admission or at least three outpatient diagnoses of T2DM based on the ICD-9-CM codes: 250.0, 250.1, 250.2, 250.3, 250.4, 250.5, 250.6, 250.7, 250.8, and 250.9 combined with treated with insulin or diabetes- specific hypoglycemic agents for > 90 cumulative defined daily doses (DDDs) within 365 days.

### Covariate assessment and adjustment

Patients’ demographics, baseline comorbidities, and use of co-medications were identified as covariates. We used outpatient files to ascertain whether study subjects had comorbidities. Comorbidities were determined in a patient if he or she was diagnosed for any of the aforementioned diseases on at least two outpatient claims during the study period. In addition, data on the use of concomitant medications were extracted from The NHIRD prescription database by using the code of the ATC classification system.

### Statistical analysis

Chi-square and t-tests were used to evaluate the differences in distributions of categorical and continuous variables between the study cohorts. In addition, we used the Kaplan–Meier method to estimate the cumulative incidence of T2DM for study cohorts. The log-rank test was used to evaluate differences in cumulative incidence of T2DM between the cohorts. Furthermore, the multivariable Cox proportional hazards regression models were performed to compute hazard ratios (HRs) with 95% confidence intervals (CIs) to assess the association of use of lansoprazole and the risk of incident T2DM after adjusting for potential confounders.The log minus log plot of survival was used to verify that explanatory variables analyzed satisfy the proportionality assumption of the Cox regression model [23]. All statistical tests were two-sided, and a level of 0.05 was considered statistically significant. All data analyses were performed using SAS software, version 9.1 (SAS Institute, Cary, NC).

## Results

Table 1 shows the distributions of age, sex, baseline comorbidities, and use of concomitant medications in the lansoprazole-exposed and comparison cohorts. There were no significant statistical differences in the distributions of age, sex, and concomitant medications after the propensity score matching schemes.

**Table 1:** Baseline characteristics of study cohorts

Variable	Study cohorts		p value
	Other PPIs (N = 3336)	Lansoprazole (N = 1668)	
Age (mean ± SD)	51.06 ± 14.77	52.20 ± 14.51	0.009
Gender (No., %)			1.000
Female	1316(39.4)	658(39.4)	
Male	2020(60.6)	1010(60.6)	
Comorbidities (No., %)			
Heart failure	136(4.1)	53(3.2)	0.116
Malignant neoplasms	425(12.7)	234(14.0)	0.204
Hyperlipidemia	524(15.7)	249(14.9)	0.472
Cardiovascular disease	654(19.6)	327(19.6)	1.000
Hypertension	1260(37.8)	630(37.8)	1.000
Chronic liver disease	776(23.3)	431(25.8)	0.044
Chronic kidney disease	46(1.4)	23(1.4)	1.000
Co-medications (No., %)			
Thiazide	118(3.5)	48(2.9)	0.219
Beta blocking agents	907(27.2)	433(26.0)	0.355
Statins	422(12.6)	211(12.6)	1.000
Corticosteroids	1183(35.5)	609(36.5)	0.466

**Fig. 2:**
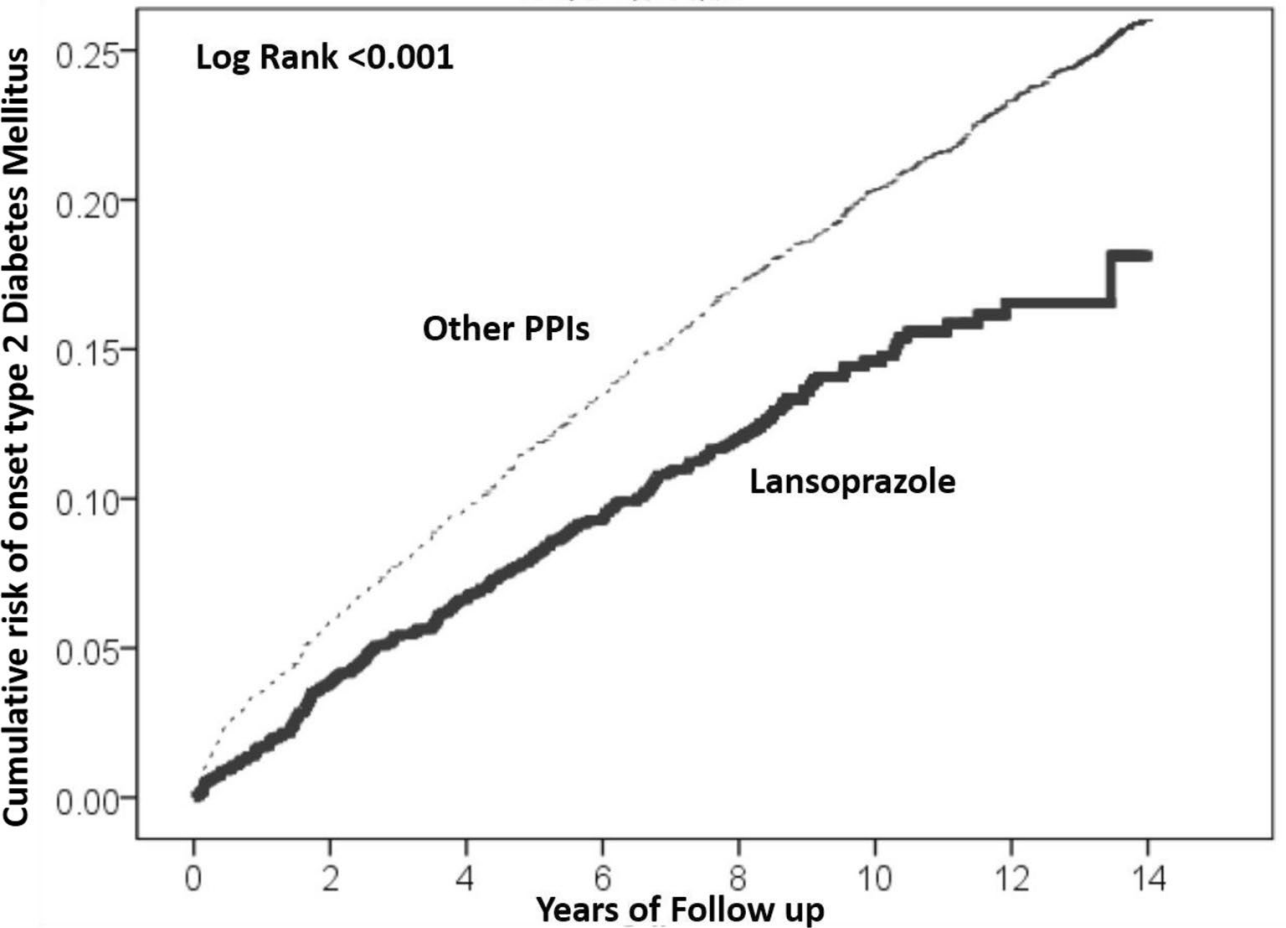
Kaplan–Meier curves for the cumulative risk of incident coronary heart disease stratified by administration of Lansoprazole and other PPI with log-rank test

In the follow-up period of 14931.33 person-years among patients received lansoprazole, there were 235 newly diagnosed T2DM, with an incidence rate of 157.39 per 10,000 person-years. Comparatively, there were 845 incident T2DM among 38529.93person-years in the comparison cohort with exposure to other PPIs, with an incidence rate of 219.31 per 10,000 person-years. The Kaplan–Meier curves for the cumulative incidence of T2DM among the two cohorts are shown in Fig. 2. The cumulative incidence of T2DM was significantly higher in the comparison cohort with exposure to other PPIs than in the cohort with prescription of lansoprazole (p < 0.001).

Table 2 presents the association between use of lansoprazole versus use of other PPIs and the risk of T2DM. Patients received lansoprazole treatments had significantly reduced risk of T2DM as compared with those who received other PPIs treatments, with an adjusted HR of 0.65 (95% confidence interval, 0.56–0.76). More interestingly, as shown in Table 3, the inverse association between use of lansoprazole and risk of T2DM was observed in both men and women and in various age groups.

**Table 2:** Association between prescription of lansoprazole and risk of type 2 diabetes mellitus (T2DM)

Variable	No. of subjects	No. of T2DM cases	Crude HR (95% CI)	Adjusted HR (95% CI)
Othe PPIs	3336	845	1.00	1.00
Lansoprazole	1668	235	0.69 (0.59–0.80)	0.65 (0.56–0.76)

Hazard ratios were adjusted for age, sex, index date, comorbidities, including heart failure, malignant neoplasms, cardiovascular disease, hypertension, hyperlipidemia, chronic liver disease, and chronic kidney disease as well as use of concomitant medications, including thiazide, beta blocking agents, statins, and corticosteroids

*T2DM* type 2 diabetes mellitus, *HR* hazard ratio, *CI* confidence interval, *PPI* Proton pump inhibitor

**Table 3:** Association between prescription of lansoprazole and risk of type 2 diabetes mellitus (T2DM) stratified by sex and age

Variable	No. of subjects	No. of CHD cases	Crude HR (95% CI)	Adjusted HR (95% CI)
Gender				
Males				
Othe PPIs	2020	469	1.00	1.00
Lansoprazole	1010	128	0.68 (0.56–0.84)	0.66 (0.53–0.82)
Females				
Othe PPIs	1316	376	1.00	1.00
Lansoprazole	658	107	0.69 (0.55–0.86)	0.66 (0.53–0.82)
Age (years)				
20–39				
Othe PPIs	716	77	1.00	1.00
Lansoprazole	358	17	0.60 (0.35–1.02)	0.52 (0.30–0.89)
40–59				
Othe PPIs	1536	417	1.00	1.00
Lansoprazole	768	118	0.73 (0.59–0.90)	0.71 (0.58–0.88)
≥60				
Othe PPIs	1084	351	1.00	1.00
Lansoprazole	542	100	0.65 (0.52–0.82)	0.64 (0.51–0.81)

Hazard ratios were adjusted for age, sex, index date, comorbidities, including heart failure, malignant neoplasms, cardiovascular disease, hypertension, hyperlipidemia, chronic liver disease, and chronic kidney disease as well as use of concomitant medications, including thiazide, beta blocking agents, statins, and corticosteroids

*T2DM* type 2 diabetes mellitus, *HR* hazard ratio, *CI* confidence interval, *PPI* proton pump inhibitor

## Discussion

In the current retrospective cohort study, our results showed that clinical use of lansoprazole was associated with a significantly reduced risk of T2DM. More importantly, the inverse association between treatment of lansoprazole and the risk of T2DM has been shown consistently in both genders and across different age groups.

Lansoprazole is one of the most commonly prescribed drugs over the past few decades and is an effective PPI that is widely used for gastric acid-related disorders because of its ability to reduce acid secretion of parietal cells [24, 25]. Lansoprazole has been reported to inhibit inflammation, oxidative stress, and growth of cancer cells [17, 18, 26, 27]. More importantly, clinical research and animal model studies have indicated that lansoprazole was associated with improved glycemic control, lowered HbA1c levels, and increased circulating insulin concentration. It has been noted that adipose tissues play central roles in glucose and lipid homeostasis [28]. In particular, studies conducted by Benchamana demonstrated that lansoprazole influences differentiation and function of cultured adipocytes and supports drug repositioning of lansoprazole an alternative agent for lowering blood glucose [29]. In agreement with these notions, this study based on follow-up of an Asian population demonstrated that lansoprazole was associated with reduced risk of T2DM as compared with other PPIs. Benefits from adipogenesis, anti-inflammatory and increased gastrin levels for lansoprazole, are the possible mechanisms that lansoprazole could reduce the risk of T2DM.

There are some strengths of this study. The present study used a comprehensive prescription database rather than self-reported records, thereby minimizing recall bias. In addition, the NHIRD covers a highly representative sample of Taiwan’s general population because the reimbursement policy is universal and operated by a single-payer. This allowed us to conduct our analyses in a real-life setting in an unselected patient population. Nevertheless, the rsults of this study need to be interpreted carefully because of some existing limitations. Crucial laboratory parameters, such as fasting plasma glucose and glycated hemoglobin levels were not available in the NHIRD. Accordingly, it’s not possible to evaluate the impact of lansoprazole treatments on biochemical profiles of patients with T2DM. In addition, studies that are based on medical claims data are often biased because the information on confounders contained in claims dataset is often limited [30]. Because the NHIRD includes only the claims data of patients, information on some potential confounders that are associated with T2DM risk, such as patients’ lifestyles, family history of diabetes, obesity, and genetic profiles, were not factored for analyses. If these potential confounders differentially distributed between the exposed and comparison cohorts, it cannot be ruled out that there may be residual confounding in the present study. Furthermore, the use of prescription database in this study did not permit confirmation of actual usage, as it was impossible to contact patients directly because of the anonymity of the records. The possibility of some degrees of treatment non-compliance should be also considered.

## Conclusion

The present retrospective cohort study findings observed that clinical use of lansoprazole was associated with a 35% reduced risk of type 2 diabetes relative to other PPIs. In addition, the reduced risk of T2DM associated with lansoprazole use was evident in both genders and in various age groups. Further studies are needed to determine the clinical implications of the present study.

## Data Availability

Data are available from the NHIRD published by the Taiwan National Health Insurance Administration. Due to legal restrictions imposed by the government of Taiwan concerning the Personal Information Protection Act, data cannot be made publicly available. Requests for data can be sent as a formal proposal to the NHIRD (http://www.mohw.gov.tw).
